# Elevated serum LDL-C increases the risk of Lewy body dementia: a two-sample mendelian randomization study

**DOI:** 10.1186/s12944-024-02032-0

**Published:** 2024-02-08

**Authors:** Pengdi Liu, Jin Liu, Yafei Zhang, Xin Xing, Le Zhou, Jianqiang Qu, Xianxia Yan

**Affiliations:** 1 Department of Neurosurgery, the Second Affiliated Hospital of Xi’an Jiaotong University, Xi’an, Shaanxi Province 710004 China; 2 Department of Otolaryngology Head and Neck Surgery, Xijing Hospital of Air Force Medical University, Xi’an, Shaanxi Province 710032 China; 3 Department of Hepatobiliary and Pancreatic Surgery, the First Affiliated Hospital of Zhengzhou University, Zhengzhou, Henan Province 450052 China; 4 Department of Cardiology, the Second Affiliated Hospital of Xi’an Jiaotong University, Xi’an, Shaanxi Province 710004 China

**Keywords:** Serum lipids, Lewy body dementia, Mendelian randomization, Causality

## Abstract

**Background:**

Lewy body dementia (LBD) ranks second among prevalent neurodegenerative dementias. Previous studies have revealed associations of serum lipid measures with several neurodegenerative diseases. Nevertheless, the potential connection between serum lipids and LBD remains undetermined. In this study, Mendelian randomization (MR) analyses were carried out to assess the causal relationships of several serum lipid measures with the risk of developing LBD.

**Methods:**

Genome-wide association study (GWAS) data for serum lipids and LBD in European descent individuals were acquired from publicly available genetic summary data. A series of filtering procedures were conducted to identify the genetic variant candidates that are related to serum lipids, including high-density lipoprotein cholesterol (HDL-C), low-density lipoprotein cholesterol (LDL-C), and triglycerides (TG). The causal effects were primarily determined through inverse-variance weighting (IVW)-based analyses.

**Results:**

Neither TG (odds ratio [OR] = 1.149; 95% confidence interval [CI], 0.887–1.489; *P* = 0.293) nor HDL-C (OR = 0.864; 95% CI, 0.718–1.041; *P* = 0.124) had causal effects on LBD. However, a causal relationship was identified between LDL-C and LBD (OR = 1.343; 95% CI, 1.094–1.649; *P* = 0.005), which remained significant (OR = 1.237; 95% CI, 1.015–1.508; *P* = 0.035) following adjustment for HDL-C and TG in multivariable MR.

**Conclusions:**

Elevated serum LDL-C increases the risk of LBD, while HDL-C and TG have no significant causal effects on LBD.

**Supplementary Information:**

The online version contains supplementary material available at 10.1186/s12944-024-02032-0.

## Introduction

LBD patients often exhibit several histopathological and clinical symptoms that are similar to those of both Alzheimer’s disease (AD) and Parkinson’s disease (PD) patients. The lack of specific biomarkers for precise diagnosis frequently leads to misdiagnosis or underdiagnosis of LBD [1]. Currently, there is no curative treatment available for LBD, and the primary focus of clinical management is symptoms alleviation. However, it is important to noted that several pharmacologic agents commonly used for other forms of dementia may actually exacerbate LBD symptoms [2]. As a result, LBD imposes a significant social and economic burden, surpassing that of AD [3]. Given these circumstances, identifying potential risk factors associated with LBD for preventative measures could be a feasible approach to controlling the disease.

HDL-C, LDL-C, and TG are commonly utilized clinical indicators and are readily obtainable. Serum lipids have been shown to be associated with multiple neurodegenerative diseases (e.g., AD [4] and PD [5]). Nevertheless, few studies have examined the relationships among serum lipids and LBD. A case-control study enrolling 65 LBD patients and 110 adult controls revealed associations between LBD development and levels of LDL-C and HDL-C [6]. However, conventional observational studies are inherently flawed due to their nonrandomized designs, making them susceptible to both reverse causality and residual confounding [7]. As of now, there are no large-scale, multicentre randomized controlled trials (RCTs) available. Therefore, based on existing evidence, the exact relationship between serum lipids and LBD cannot be determined.

Mendelian randomization (MR) involves the use of single-nucleotide polymorphisms (SNPs) as genetic variants. Genotype formation, occurring prior to disease onset, is usually unaffected by clinical factors. This characteristic makes MR designs highly reliable, as they are largely independent of reverse causality and confounding factors [8]. As a result, MR designs are broadly utilized to examine the causal relationships among exposures and clinical outcomes [9]. Considering the uncertain relationship between serum lipids and LBD, multivariable MR (MVMR) and univariable MR (UVMR) analyses were performed using summary-level statistics of GWASs. This study assessed causal effects of three different exposures (HDL-C, LDL-C, and TG) on LBD. Findings obtained from this study have the potential to offer novel insights into the risk factors associated with LBD, which can contribute to the improvement of LBD screening, prevention, and the optimization of clinical management strategies for LBD patients.

## Materials and methods

### Study design

The causal relationships among HDL-C, LDL-C, TG and LBD were evaluated through MR method. The robust design of MR design was built on three fundamental assumptions [10]: (I) strong associations among genetic variants and target exposures; (II) no associations among genetic variants and potential confounding factors; (III) genetic variants influence outcomes exclusively via the exposures of interest.

### Sources of GWAS data

The GWAS summary data for HDL-C, LDL-C, and TG in individuals of European descent were obtained from the OpenGWAS database. Exposure GWAS included LDL-C data for 70,814 participants, HDL-C data for 77,409 participants, and TG data for 78,700 participants. Each of the three exposure datasets had approximately 7.89 million SNPs.

LBD GWAS data were obtained from a multicentre case-control study [11] comprising 6618 individuals of European descent, including 2591 LBD patients and 4027 neurologically healthy control individuals. This study includes approximately 7.59 million SNPs. Detailed demographic characteristics, study sites/consortia, diagnostic criteria, and quality control are also available. For more detailed information on the GWAS datasets, please refer to Supplementary Table 1.

### Selection of genetic instruments

Multiple steps were executed to identify the eligible genetic variants. First, SNPs associated with three exposure factors (TG, HDL-C, LDL-C) were identified by applying a rigorous threshold of *P* < 5 × 10^− 8^. Subsequently, SNPs in linkage disequilibrium (LD, window size = 10,000 kilobase and r^2^ threshold = 0.001) were discarded. Second, the F statistics were computed to prevent weak instrument involvement. The SNPs were retained if they had F statistics greater than 10. SNP candidates were then extracted from LBD GWAS data excluding those with *P* < 5 × 10^− 6^. Proxy SNPs were determined by utilizing the “TwoSampleMR” package at the LD threshold r^2^ > 0.80. Third, SNPs absent in the outcome or lacking proper proxy SNPs were excluded from further analysis. The fourth step involved a harmonization process to exclude the SNPs that are palindromic or have inconsistent alleles between the outcome and exposure GWAS data. Finally, outlier SNPs were eliminated using the MR pleiotropy residual sum and outlier (MR-PRESSO) method [12]. Detailed information about the SNPs utilized as genetic instruments is provided in Supplementary Tables 2–5.

### MR analyses

“MendelianRandomization” (v.0.6.0) and “TwoSampleMR” (v.0.5.6) packages were used in R (v.4.2.2) for statistical analyses. Four MR methods were employed for UVMR analyses: weighted median, MR Egger, inverse-variance weighting (IVW), and weighted mode. IVW served as the primary analyses [13]. The other three MR analytical methods were conducted to complement the IVW estimates. Considering the clinical associations among the three exposures, MVMR analyses were conducted to evaluate their independent effects on LBD. IVW and MR Egger methods were used. Similarly, IVW served as the primary method. *P* < 0.05 was considered significant. Statistical power was calculated using an online tool [14] (https://shiny.cnsgenomics.com/mRnd/).

### Quality control of MR estimation

Various methods were employed to assess possible violation of the MR assumptions. Heterogeneity was identified through the Cochran Q-test [15] and funnel plot. A symmetric funnel plot is indicative of a low risk of heterogeneity. MR‒Egger intercepts [16] were utilized for directional pleiotropy detection. Leave-one-out analyses were carried out to explore the influences of any individual SNP on the overall MR estimate. In addition, MR Steiger directionality test [17] was used to explore reverse causation.

This study also explored the genetic instruments associated with several common risk factors for LBD, such as smoking [18], cancer, stroke, and coffee consumption [19]. To achieve this, the PhenoScanner database was employed, which provides comprehensive information on human genotype-phenotype associations. Following this, additional MR analyses were performed after removing these SNPs to ensure robust results.

## Results

### Genetic instruments

**Fig. 1:**
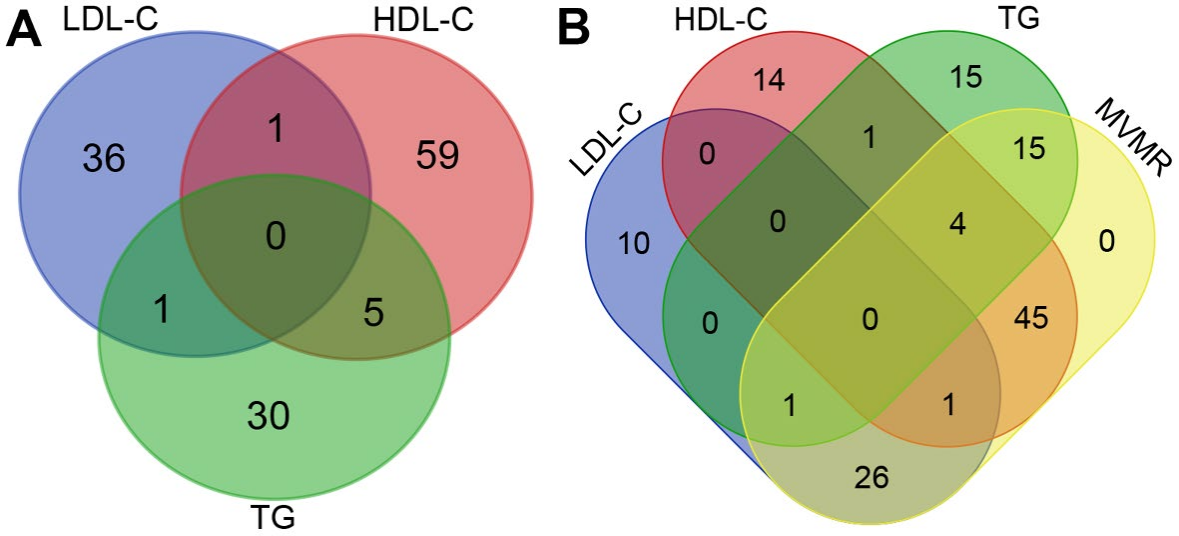
Venn diagram of eligible SNPs associated with HDL-C, LDL-C, and TG (**A**); Venn diagram of eligible SNPs utilized in UVMR and MVMR analyses (**B**)

After rigorous filtration steps, a total of 38, 65, and 36 SNPs were used in the UVMR analysis to genetically predict LDL-C, HDL-C, and TG, respectively. Notably, SNP rs28456 has associations with both HDL-C and LDL-C. Five SNPs (rs144503444, rs145947882, rs116843064, rs11231693, and rs147233090) have associations with both TG and HDL-C. Moreover, SNP rs112875651 has associations with both TG and LDL-C (Fig. 1). No F statistics were below 10. After clumping and harmonizing candidate SNPs of three exposure factors together, a total of 92 SNPs were utilized in the MVMR analyses (Supplementary Table 5). Proxy SNPs used in this study were shown in Table 1. No outlier SNPs were detected.

**Table 1:** Proxy SNPs used in this study

Target SNPs	Proxy SNPs
rs72926990	rs72928934
rs71556736	rs34430945
rs7395581	rs4752980
rs11218738	rs3937026
rs12898210	rs4775033
rs10159255	rs2131925
rs326222	rs7120957

### UVMR estimates

**Fig. 2:**
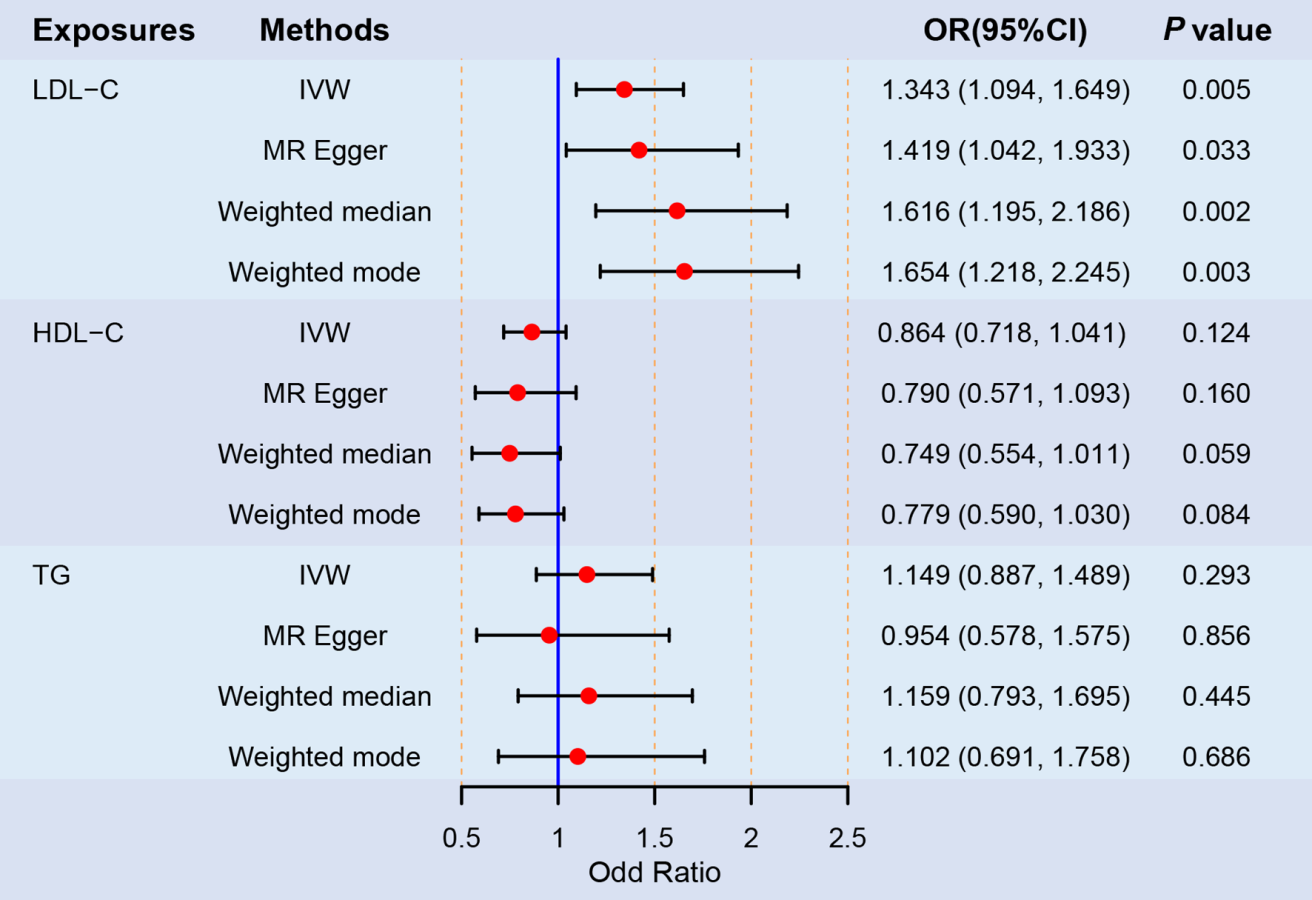
Forest plot of univariable MR analyses for the causal effects of HDL-C, LDL-C, and TG on the risk of LBD

**Fig. 3:**
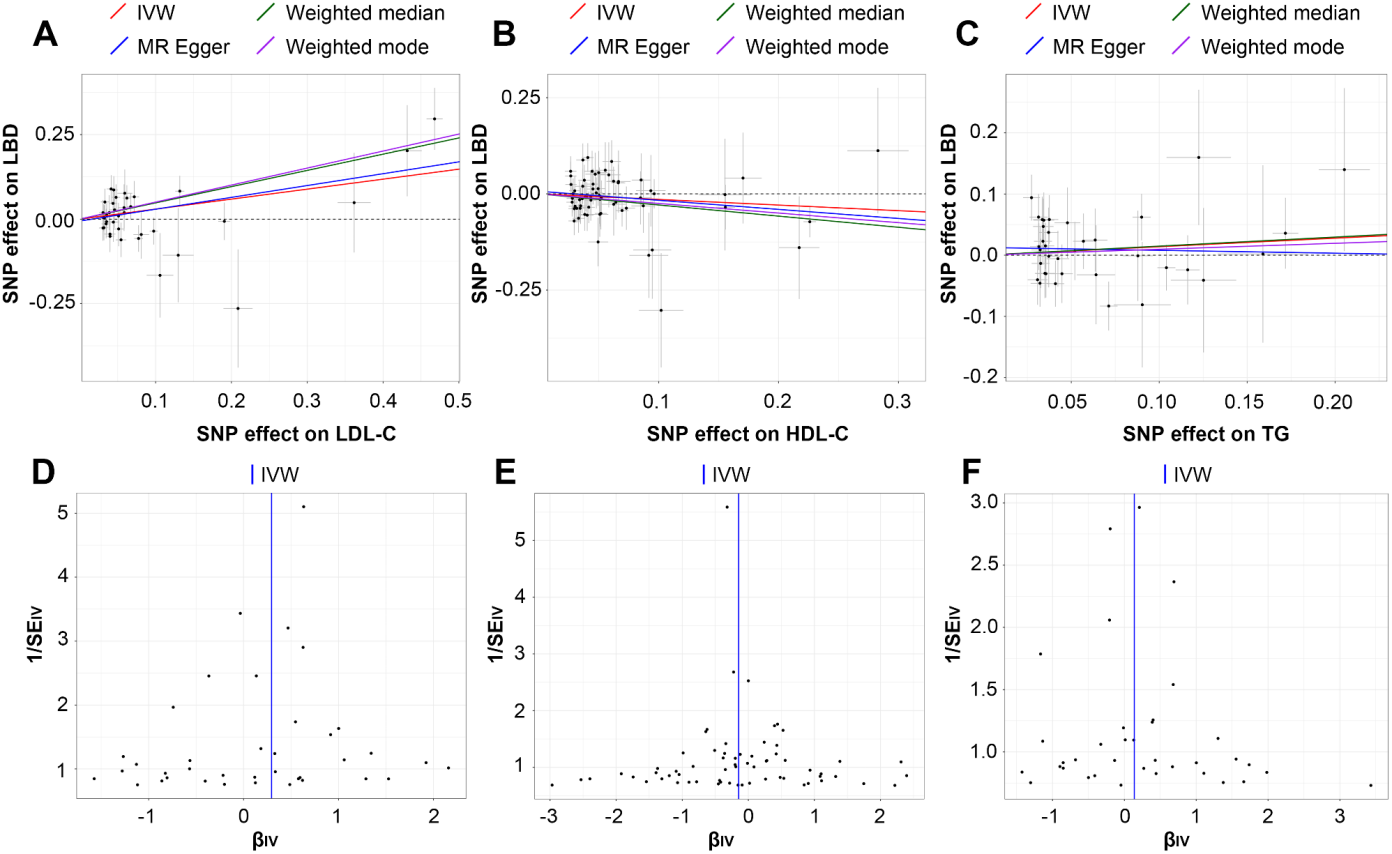
Scatter plots and funnel plots of MR analyses. Scatter plots of genetic effects on LDL-C (**A**), HDL-C (**B**), and TG (**C**) versus their effects on LBD; funnel plots of MR analyses between serum lipids (LDL-C [**D**], HDL-C [**E**], TG [**F**]) and LBD

A causal relationship between LBD and LDL-C was identified with strong evidence. IVW analysis revealed that the increased LDL-C level could significantly elevate LBD risk (OR = 1.343; 95% CI, 1.094–1.649; *P* = 0.005). Causal estimates of UVMR were broadly consistent in magnitude and direction across various MR models (Figs. 2 and 3A). The MR Steiger directionality test did not indicate any evidence of reverse causality (Supplementary Table 6). Therefore, the causal direction from LDL-C to LBD is reliable. Conversely, the causal effects of TG (OR = 1.149; 95% CI, 0.887–1.489; *P* = 0.293) and HDL-C (OR = 0.864; 95% CI, 0.718–1.041; *P* = 0.124) on LBD were not statistically significant across all MR models (Figs. 2 and 3B-C). Statistical power was presented in Supplementary Table 7. MR analyses in this study exhibited neither heterogeneity nor pleiotropy (Table 2). Additionally, the funnel plot was basically symmetrical for all analyses (Fig. 3D-F). Furthermore, leave-one-out analyses reaffirmed the reliability of the causal effects (Supplementary Fig. 1).

**Table 2:** Heterogeneity and pleiotropy analyses

Exposures	Heterogeneity			Pleiotropy	
	Q	*P*		Egger intercept	*P*
**LDL-C**	43.154	0.225		-0.006	0.642
**HDL-C**	59.810	0.625		0.007	0.511
**TG**	35.599	0.440		0.013	0.402

### MVMR estimates

**Fig. 4:**
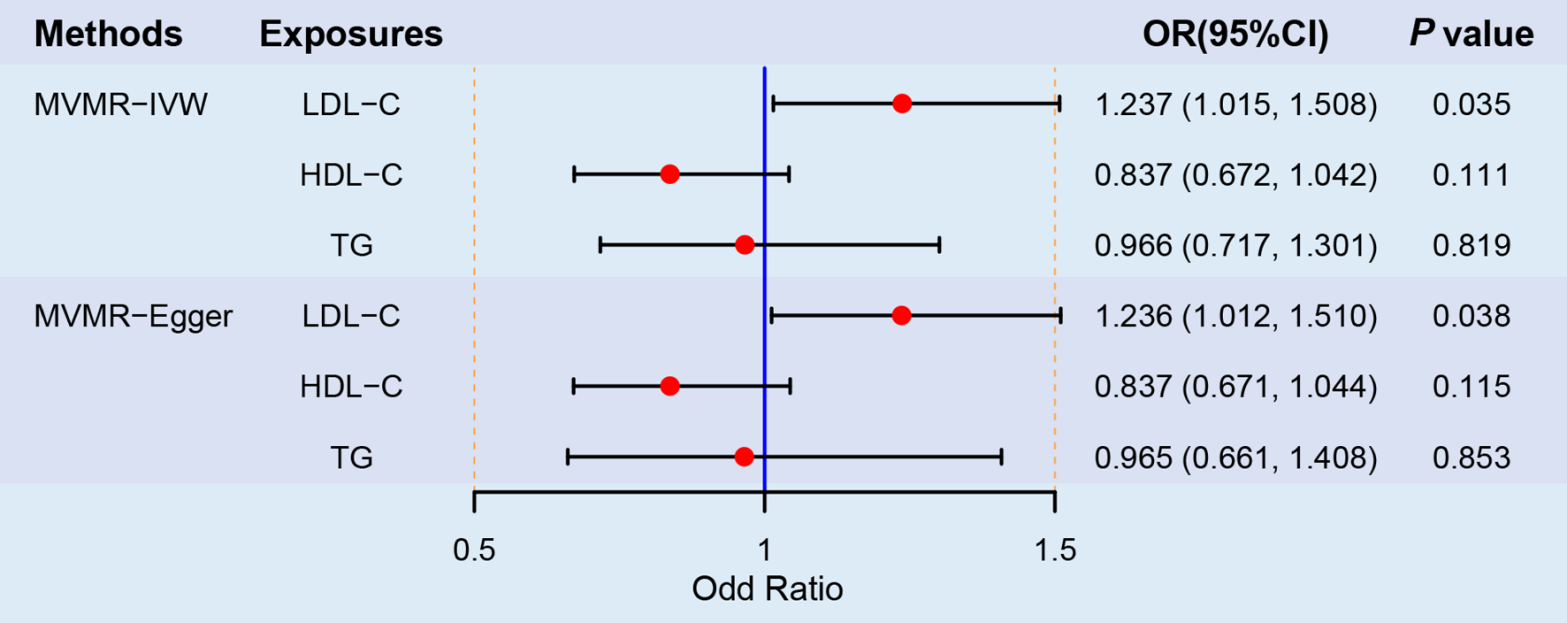
Forest plot of multivariable MR analyses for the causal effects of LDL-C on the risk of LBD adjusting for HDL-C and TG

In MVMR analyses, the detrimental effects of LDL-C on LBD remained significant after adjustment for TG and HDL-C, with consistent estimates across IVW (OR = 1.237; 95% CI, 1.015–1.508; *P* = 0.035) and MR Egger (OR = 1.236; 95% CI, 1.012–1.510; *P* = 0.038) (Fig. 4). Causal effects of TG and HDL-C on LBD were not observed in MVMR analyses (Fig. 4). No heterogeneity was detected (*P* = 0.637). The MR‒Egger intercept (intercept < 0.001; SE = 0.007; *P* = 0.993) suggest no directional pleiotropy exists in MVMR analyses.

### Confounding analyses

Although no heterogeneity or pleiotropy was detected, the secondary traits (cancer, stroke, coffee consumption, and smoking) of SNPs utilized in this MR analyses were further manually checked. The HDL-C-associated SNPs had no associations with any confounders. For LDL-C, rs635634 was associated with invasive ovarian cancer and ischaemic stroke; rs140244541 was associated with smoking. For TG, rs1260326 was associated with coffee consumption. After removing these SNPs, the causality between the three exposure factors (LDL-C, HDL-C, and TG) and LBD remained unchanged (Supplementary Table 8).

## Discussion

The aetiology and risk factors for LBD remain unclear, hindering the development of prevention strategies. Serum lipids are commonly used as indicators in health screenings, and accumulating evidence has revealed their associations with the potential risk of dementia, particularly AD [20–22]. However, limited evidence exists regarding the causal roles of serum lipids in LBD. To further examine whether serum lipids influence the LBD risk, MR design was applied in this study to assess their causal effects, and the results demonstrated a causal relationship between higher serum LDL-C levels and an increased risk of LBD, while HDL-C or TG did not impact the risk of LBD.

LBD, AD, and PD are prevalent neurodegenerative diseases. They exhibit overlapping symptom profiles, similar pathological manifestations of the deposition of α-synuclein, and even share potential genomic risk loci [23, 24]. However, the risk factors for these diseases are not consistently aligned. Smoking can significantly increase the risk of AD through smoking-related cerebral oxidative mechanisms [25]. Conversely, research suggests that smoking may have protective effects on PD [26] and is associated with a reduced risk of Lewy-related pathology [18]. Education exhibits opposing effects on the risk of AD and PD [27, 28], but shows no correlation with the risk of LBD [19]. Regarding serum lipids, some multicentre cohort studies with large sample sizes have reported that increased LDL-C attenuates the risk of PD, while HDL-C and TG show no association with PD risk [5, 29]. In contrast, multiple meta-analyses have revealed that high LDL-C levels increase the AD risk [4, 30]. These results highlight the diverse roles of LDL-C in neurodegenerative diseases. However, research on the roles of LDL-C in LBD is limited. A single-centre observational study reported the association of elevated serum LDL-C and reduced HDL-C with an increased LBD risk [6], which aligns with the MR analysis results of LDL-C in this study but not those of HDL-C. The relationship between HDL-C and LBD was not statistically significant in this study, although the OR values of HDL-C were less than 1 and demonstrated consistent estimations across various MR methods. The MR methods in this study, in comparison with conventional observational designs, could effectively minimize bias and enhance the credibility of the findings.

The underlying mechanisms of the causality between serum LDL-C and LBD are complex. In the periphery, serum cholesterol typically binds to circulating lipoproteins, facilitating its solubility and transportation to various tissues. Low-density lipoprotein (LDL) carries the majority of cholesterol. A vital role for cholesterol is to maintain neuronal structure and function. Under normal circumstances, blood-brain barrier (BBB) restricts the passage of serum cholesterol [31]. However, elevated serum cholesterol increases BBB permeability, allowing peripheral cholesterol, particularly LDL-C, to enter the brain and disrupt cholesterol metabolism [32]. Moreover, LDL binds to the LDL receptor on the BBB and is transported into the central nervous system via transcellular processes, suggesting a potential mechanism for the transportation of LDL from the periphery to the brain [33].

Evidence indicates that dysfunction in central cholesterol metabolism is closely linked to neurodegenerative pathologies [34]. Brain cholesterol plays a role in facilitating interactions between α-synuclein oligomers [35] and initiates their clustering [36]. Cholesterol-rich regions can serve as sites for the aggregation of α-synuclein [37]. Despite numerous studies investigating the possible involvement of cholesterols in the pathological process of dementia, the exact mechanisms underlying the causality between serum LDL-C and LBD remain unclear and demand further investigation.

### Strengths and limitations

The current study has several noteworthy strengths. First, it is the first comprehensive MR study examining the causal relationships between serum lipids and LBD, providing a valuable reference for subsequent research in this area. Second, in the absence of RCTs, this MR analysis serves as a significant alternative study method, capitalizing on the inherent advantages of the MR design. Third, rigorous quality control measures were employed, and no evidence of heterogeneity, pleiotropy, or violation of MR assumptions was found, ensuring the reliability of the study.

Certain limitations are noted in this study. First, the GWAS data utilized are derived from participants of European ancestry, limiting the direct generalization of findings in this study to other populations. Second, while a causal relationship between LBD and LDL-C is indicated, the underlying molecular mechanisms remain incompletely understood. Third, due to the absence of detailed information on variables such as sex, age, LBD subtypes, and other clinical features in the summary-level data, subgroup analysis to stratify the population into different subgroups was not feasible.

## Conclusions

This MR study offers compelling evidence that elevated serum LDL-C increases the risk of LBD. However, HDL-C and TG exhibit no significant causal effects on LBD. The findings of this study underscore the need to pay more attention to individuals with high serum LDL-C during the process of LBD screening and prevention, and contribute to the identification of feasible clinical management strategies for LBD patients.

### Electronic supplementary material

Below is the link to the electronic supplementary material.

**Supplementary Material 1: Supplementary Fig. 1** Leave-one-out analyses of MR estimates of genetic risk of LDL-C (**A**), HDL-C (**B**), and TG (**C**) on LBD

**Supplementary Material 2: Supplementary Table 1** Detailed information of GWAS datasets in the current study.

**Supplementary Material 3: Supplementary Table 2** Eligible genetic instruments associated with LDL-C.

**Supplementary Material 4: Supplementary Table 3** Eligible genetic instruments associated with HDL-C.

**Supplementary Material 5: Supplementary Table 4** Eligible genetic instruments associated with TG.

**Supplementary Material 6: Supplementary Table 5** Eligible genetic instruments used in MVMR.

**Supplementary Material 7: Supplementary Table 6** Steiger direction test from LDL-C to LBD.

**Supplementary Material 8: Supplementary Table 7** Statistical power calculation for MR analyses.

**Supplementary Material 9: Supplementary Table 8** IVW MR analyses after removing SNPs with secondary traits.

## Data Availability

The datasets generated or analysed during the current study are available in OpenGWAS database (https://gwas.mrcieu.ac.uk/).

## Abbreviations

AD: Alzheimer’s disease
CI: confidence interval
HDL-C: high-density lipoprotein cholesterol
IVW: Inverse-variance weighting
LBD: Lewy body dementia
LDL-C: low-density lipoprotein cholesterol
MR: Mendelian randomization
OR: odds ratio
PD: Parkinson’s disease
TG: triglycerides
