# Supplementary figures and images for: Elevated serum LDL-C increases the risk of Lewy body dementia: a two-sample mendelian randomization study
Source: Lipids Health Dis. 2024 Feb 8;23:42. doi: 10.1186/s12944-024-02032-0 (PMC10851540; doi:10.1186/s12944-024-02032-0)

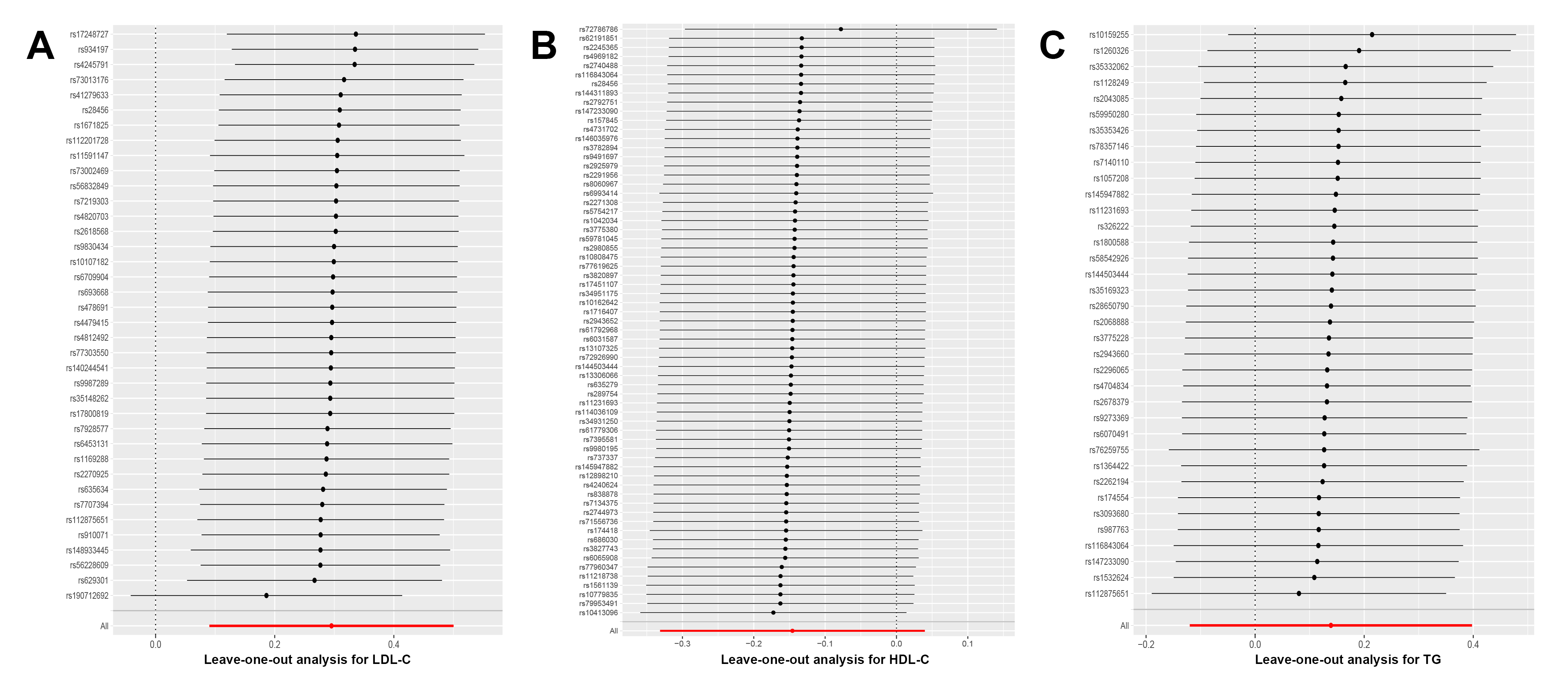

Supplement: Supplementary file 1 — Supplementary Material 1: Supplementary Fig. 1 Leave-one-out analyses of MR estimates of genetic risk of LDL-C (A), HDL-C (B), and TG (C) on LBD [file 12944_2024_2032_MOESM3_ESM.tif]
