# Supplementary material for: Elevated serum LDL-C increases the risk of Lewy body dementia: a two-sample mendelian randomization study
Source: Lipids Health Dis. 2024 Feb 8;23:42. doi: 10.1186/s12944-024-02032-0 (PMC10851540; doi:10.1186/s12944-024-02032-0)
Supplement: Supplementary file 2 — Supplementary Material 2: Supplementary Table 1 Detailed information of GWAS datasets in the current study. [file 12944_2024_2032_MOESM4_ESM.docx]

**Supplementary Table 1**

Detailed information of GWAS datasets in the current study.

| Phenotype | Consortium | Ancestry | Participants | Note | Web source |
| --- | --- | --- | --- | --- | --- |
| LDL cholesterol | Within family GWAS consortium | European | 70814 | Population estimate | <https://gwas.mrcieu.ac.uk/> |
| HDL cholesterol | Within family GWAS consortium | European | 77409 | Population estimate | <https://gwas.mrcieu.ac.uk/> |
| Triglycerides | Within family GWAS consortium | European | 78700 | Population estimate | <https://gwas.mrcieu.ac.uk/> |
| Lewy body dementia | NA | European | 6618 | Population estimate  PMID: 33589841 | <https://gwas.mrcieu.ac.uk/> |
