# Supplementary material for: Elevated serum LDL-C increases the risk of Lewy body dementia: a two-sample mendelian randomization study
Source: Lipids Health Dis. 2024 Feb 8;23:42. doi: 10.1186/s12944-024-02032-0 (PMC10851540; doi:10.1186/s12944-024-02032-0)
Supplement: Supplementary file 3 — Supplementary Material 3: Supplementary Table 2 Eligible genetic instruments associated with LDL-C. [file 12944_2024_2032_MOESM5_ESM.docx]

**Supplementary Table 2**

Eligible genetic instruments associated with LDL-C.

| SNP | Effect allele | Other allele | Beta | SE | *p* value |
| --- | --- | --- | --- | --- | --- |
| rs10107182 | T | C | -0.0352 | 0.0054 | 4.98E-11 |
| rs112201728 | T | C | 0.0810 | 0.0103 | 3.64E-15 |
| rs112875651 | A | G | -0.0621 | 0.0052 | 1.20E-32 |
| rs11591147 | T | G | -0.3619 | 0.0217 | 3.08E-62 |
| rs1169288 | C | A | 0.0331 | 0.0055 | 1.51E-09 |
| rs140244541 | A | G | -0.0389 | 0.0070 | 2.71E-08 |
| rs148933445 | A | G | -0.4319 | 0.0191 | 5.50E-113 |
| rs1671825 | C | T | -0.1058 | 0.0176 | 1.83E-09 |
| rs17248727 | C | T | -0.1906 | 0.0079 | 3.98E-129 |
| rs17800819 | T | C | -0.0459 | 0.0074 | 5.15E-10 |
| rs190712692 | A | G | -0.4680 | 0.0106 | 1.00E-200 |
| rs2270925 | C | T | 0.0438 | 0.0053 | 1.64E-16 |
| rs2618568 | A | C | -0.0306 | 0.0053 | 1.06E-08 |
| rs28456 | G | A | -0.0399 | 0.0055 | 3.40E-13 |
| rs35148262 | G | A | 0.0306 | 0.0051 | 2.60E-09 |
| rs41279633 | T | G | 0.0544 | 0.0069 | 3.49E-15 |
| rs4245791 | T | C | -0.0775 | 0.0057 | 1.40E-42 |
| rs4479415 | A | G | 0.0311 | 0.0056 | 2.57E-08 |
| rs478691 | G | T | 0.0324 | 0.0052 | 3.12E-10 |
| rs4812492 | C | T | 0.0355 | 0.0051 | 3.33E-12 |
| rs4820703 | C | T | 0.0398 | 0.0072 | 3.24E-08 |
| rs56228609 | T | C | -0.0448 | 0.0054 | 8.91E-17 |
| rs56832849 | T | C | 0.0512 | 0.0067 | 3.21E-14 |
| rs629301 | T | G | 0.1319 | 0.0059 | 2.10E-110 |
| rs635634 | T | C | 0.0718 | 0.0064 | 3.99E-29 |
| rs6453131 | G | T | 0.0672 | 0.0053 | 1.82E-37 |
| rs6709904 | G | A | -0.0442 | 0.0080 | 3.07E-08 |
| rs693668 | A | G | 0.0510 | 0.0053 | 7.70E-22 |
| rs7219303 | G | A | -0.0318 | 0.0051 | 3.37E-10 |
| rs73002469 | T | C | -0.1298 | 0.0204 | 2.03E-10 |
| rs73013176 | C | T | -0.2087 | 0.0192 | 1.95E-27 |
| rs7707394 | A | G | 0.0478 | 0.0054 | 5.76E-19 |
| rs77303550 | T | C | -0.0586 | 0.0064 | 3.25E-20 |
| rs7928577 | T | G | 0.0593 | 0.0099 | 2.20E-09 |
| rs910071 | T | C | -0.0413 | 0.0055 | 4.26E-14 |
| rs934197 | A | G | 0.0975 | 0.0054 | 3.55E-73 |
| rs9830434 | C | T | 0.0347 | 0.0060 | 5.72E-09 |
| rs9987289 | G | A | 0.0584 | 0.0083 | 1.69E-12 |
