# Supplementary material for: Elevated serum LDL-C increases the risk of Lewy body dementia: a two-sample mendelian randomization study
Source: Lipids Health Dis. 2024 Feb 8;23:42. doi: 10.1186/s12944-024-02032-0 (PMC10851540; doi:10.1186/s12944-024-02032-0)
Supplement: Supplementary file 4 — Supplementary Material 4: Supplementary Table 3 Eligible genetic instruments associated with HDL-C. [file 12944_2024_2032_MOESM6_ESM.docx]

**Supplementary Table 3**

Eligible genetic instruments associated with HDL-C.

| SNP | Effect allele | Other allele | Beta | SE | *p* value |
| --- | --- | --- | --- | --- | --- |
| rs10162642 | A | G | -0.0521 | 0.0057 | 4.74E-20 |
| rs10413096 | A | G | 0.0408 | 0.0049 | 5.54E-17 |
| rs1042034 | T | C | -0.0642 | 0.0058 | 3.44E-28 |
| rs10779835 | C | T | 0.0623 | 0.0048 | 7.22E-39 |
| rs10808475 | T | C | 0.0361 | 0.0049 | 1.73E-13 |
| rs11218738 | A | G | 0.0367 | 0.0055 | 1.76E-11 |
| rs11231693 | A | G | -0.0664 | 0.0101 | 4.09E-11 |
| rs114036109 | G | A | 0.0366 | 0.0064 | 1.35E-08 |
| rs116843064 | A | G | 0.2172 | 0.0168 | 2.47E-38 |
| rs12898210 | G | T | -0.0470 | 0.0082 | 1.05E-08 |
| rs13107325 | T | C | -0.0848 | 0.0094 | 2.34E-19 |
| rs13306066 | A | G | 0.0934 | 0.0123 | 3.04E-14 |
| rs144311893 | T | C | 0.1020 | 0.0185 | 3.28E-08 |
| rs144503444 | C | T | -0.1553 | 0.0218 | 1.13E-12 |
| rs145947882 | C | A | -0.1703 | 0.0156 | 1.20E-27 |
| rs146035976 | T | C | -0.0946 | 0.0158 | 2.01E-09 |
| rs147233090 | T | C | -0.0917 | 0.0162 | 1.45E-08 |
| rs1561139 | T | G | 0.0662 | 0.0047 | 1.45E-44 |
| rs157845 | C | T | -0.0383 | 0.0055 | 4.64E-12 |
| rs1716407 | A | G | -0.0398 | 0.0050 | 2.15E-15 |
| rs174418 | C | T | -0.0962 | 0.0050 | 6.56E-83 |
| rs17451107 | C | T | 0.0279 | 0.0048 | 7.68E-09 |
| rs2245365 | A | C | 0.0294 | 0.0047 | 3.23E-10 |
| rs2271308 | C | T | 0.0302 | 0.0053 | 1.14E-08 |
| rs2291956 | T | C | 0.0508 | 0.0071 | 1.15E-12 |
| rs2740488 | C | A | -0.0694 | 0.0053 | 1.23E-39 |
| rs2744973 | T | C | -0.0379 | 0.0050 | 5.31E-14 |
| rs2792751 | C | T | -0.0403 | 0.0052 | 1.10E-14 |
| rs28456 | G | A | -0.0517 | 0.0050 | 6.12E-25 |
| rs289754 | T | C | -0.0479 | 0.0051 | 7.24E-21 |
| rs2925979 | C | T | 0.0416 | 0.0051 | 1.79E-16 |
| rs2943652 | T | C | -0.0386 | 0.0049 | 2.15E-15 |
| rs2980855 | C | T | 0.0434 | 0.0047 | 3.24E-20 |
| rs34931250 | T | C | -0.0583 | 0.0097 | 1.64E-09 |
| rs34951175 | T | C | -0.0510 | 0.0081 | 3.42E-10 |
| rs3775380 | G | A | -0.0277 | 0.0047 | 4.15E-09 |
| rs3782894 | T | G | 0.0316 | 0.0047 | 1.81E-11 |
| rs3820897 | C | T | -0.0339 | 0.0059 | 1.02E-08 |
| rs3827743 | A | G | 0.0265 | 0.0048 | 2.95E-08 |
| rs4240624 | A | G | 0.0853 | 0.0077 | 1.15E-28 |
| rs4731702 | T | C | 0.0344 | 0.0047 | 1.73E-13 |
| rs4969182 | C | T | 0.0331 | 0.0049 | 9.08E-12 |
| rs5754217 | T | G | -0.0348 | 0.0056 | 6.32E-10 |
| rs59781045 | T | C | 0.0871 | 0.0099 | 1.46E-18 |
| rs6031587 | T | C | -0.0505 | 0.0090 | 2.07E-08 |
| rs6065908 | T | C | -0.0662 | 0.0058 | 9.30E-30 |
| rs61779306 | A | G | -0.0494 | 0.0058 | 2.14E-17 |
| rs61792968 | A | G | -0.0433 | 0.0079 | 3.53E-08 |
| rs62191851 | G | A | -0.0494 | 0.0083 | 2.73E-09 |
| rs635279 | T | C | 0.0299 | 0.0047 | 2.19E-10 |
| rs686030 | A | C | 0.0444 | 0.0067 | 2.71E-11 |
| rs6993414 | G | A | 0.1556 | 0.0080 | 3.82E-84 |
| rs7134375 | A | C | 0.0266 | 0.0047 | 1.56E-08 |
| rs71556736 | T | C | 0.0499 | 0.0073 | 7.48E-12 |
| rs72786786 | A | G | 0.2260 | 0.0052 | 1.00E-200 |
| rs72926990 | A | G | -0.0286 | 0.0051 | 1.92E-08 |
| rs737337 | C | T | -0.0554 | 0.0080 | 3.89E-12 |
| rs7395581 | A | G | -0.0451 | 0.0053 | 2.24E-17 |
| rs77619625 | T | C | 0.0624 | 0.0107 | 5.87E-09 |
| rs77960347 | G | A | 0.2829 | 0.0255 | 1.32E-28 |
| rs79953491 | G | A | 0.0607 | 0.0076 | 1.39E-15 |
| rs8060967 | T | C | 0.0730 | 0.0072 | 3.32E-24 |
| rs838878 | G | A | -0.0452 | 0.0051 | 3.92E-19 |
| rs9491697 | G | A | -0.0295 | 0.0048 | 8.63E-10 |
| rs9980195 | C | T | -0.0268 | 0.0047 | 1.01E-08 |
