# Supplementary material for: Elevated serum LDL-C increases the risk of Lewy body dementia: a two-sample mendelian randomization study
Source: Lipids Health Dis. 2024 Feb 8;23:42. doi: 10.1186/s12944-024-02032-0 (PMC10851540; doi:10.1186/s12944-024-02032-0)
Supplement: Supplementary file 5 — Supplementary Material 5: Supplementary Table 4 Eligible genetic instruments associated with TG. [file 12944_2024_2032_MOESM7_ESM.docx]

**Supplementary Table 4**

Eligible genetic instruments associated with TG.

| SNP | Effect allele | Other allele | Beta | Se | *p* value |
| --- | --- | --- | --- | --- | --- |
| rs10159255 | A | C | -0.0713 | 0.0050 | 2.41E-46 |
| rs1057208 | T | C | 0.0447 | 0.0062 | 6.18E-13 |
| rs11231693 | A | G | 0.0641 | 0.0109 | 3.85E-09 |
| rs1128249 | T | G | -0.0410 | 0.0049 | 1.10E-16 |
| rs112875651 | A | G | -0.0900 | 0.0051 | 2.27E-69 |
| rs116843064 | A | G | -0.2053 | 0.0145 | 2.00E-45 |
| rs1260326 | C | T | -0.1041 | 0.0051 | 7.70E-92 |
| rs1364422 | T | C | 0.0371 | 0.0056 | 3.85E-11 |
| rs144503444 | C | T | 0.1590 | 0.0257 | 6.04E-10 |
| rs145947882 | C | A | 0.1252 | 0.0186 | 1.74E-11 |
| rs147233090 | T | C | 0.1225 | 0.0183 | 2.11E-11 |
| rs1532624 | A | C | -0.0274 | 0.0048 | 1.36E-08 |
| rs174554 | G | A | 0.0375 | 0.0051 | 2.19E-13 |
| rs1800588 | T | C | 0.0424 | 0.0064 | 2.44E-11 |
| rs2043085 | C | T | -0.0322 | 0.0053 | 1.06E-09 |
| rs2068888 | A | G | -0.0323 | 0.0050 | 7.13E-11 |
| rs2262194 | G | T | -0.0344 | 0.0059 | 4.92E-09 |
| rs2296065 | A | G | -0.0636 | 0.0069 | 1.54E-20 |
| rs2678379 | G | A | 0.0569 | 0.0059 | 6.43E-22 |
| rs28650790 | T | C | 0.0520 | 0.0068 | 2.34E-14 |
| rs2943660 | G | T | 0.0353 | 0.0050 | 1.86E-12 |
| rs3093680 | C | T | -0.0314 | 0.0051 | 5.37E-10 |
| rs326222 | C | T | 0.0326 | 0.0051 | 2.24E-10 |
| rs35169323 | T | G | -0.0372 | 0.0066 | 1.75E-08 |
| rs35332062 | A | G | -0.1162 | 0.0068 | 5.99E-65 |
| rs35353426 | T | C | 0.0308 | 0.0056 | 3.15E-08 |
| rs3775228 | T | C | 0.0316 | 0.0051 | 3.81E-10 |
| rs4704834 | G | A | 0.0339 | 0.0051 | 2.57E-11 |
| rs58542926 | T | C | -0.0879 | 0.0085 | 3.12E-25 |
| rs59950280 | A | G | 0.0356 | 0.0055 | 1.10E-10 |
| rs6070491 | T | C | -0.0341 | 0.0058 | 4.06E-09 |
| rs7140110 | C | T | 0.0350 | 0.0057 | 7.48E-10 |
| rs76259755 | C | T | -0.1718 | 0.0073 | 6.40E-124 |
| rs78357146 | G | A | -0.0904 | 0.0162 | 2.23E-08 |
| rs9273369 | C | T | -0.0478 | 0.0073 | 5.89E-11 |
| rs987763 | T | C | -0.0335 | 0.0052 | 8.63E-11 |
