# Supplementary material for: Elevated serum LDL-C increases the risk of Lewy body dementia: a two-sample mendelian randomization study
Source: Lipids Health Dis. 2024 Feb 8;23:42. doi: 10.1186/s12944-024-02032-0 (PMC10851540; doi:10.1186/s12944-024-02032-0)
Supplement: Supplementary file 6 — Supplementary Material 6: Supplementary Table 5 Eligible genetic instruments used in MVMR. [file 12944_2024_2032_MOESM8_ESM.docx]

**Supplementary Table 5**

Eligible genetic instruments used in MVMR.

| SNP | *p* value (exposure) | | |
| --- | --- | --- | --- |
|  | HDL-C | LDL-C | TG |
| rs10107182 | 4.77E-01 | 4.98E-11 | 8.52E-03 |
| rs10162642 | 4.74E-20 | 1.18E-01 | 3.37E-02 |
| rs10779835 | 7.22E-39 | 3.88E-01 | 2.31E-19 |
| rs10808475 | 1.73E-13 | 1.64E-01 | 4.10E-05 |
| rs11218738 | 1.76E-11 | 1.20E-01 | 2.76E-01 |
| rs112201728 | 7.27E-04 | 3.64E-15 | 4.31E-06 |
| rs11231693 | 4.09E-11 | 7.84E-01 | 3.85E-09 |
| rs1128249 | 1.41E-12 | 5.98E-03 | 1.10E-16 |
| rs112875651 | 1.50E-16 | 1.20E-32 | 2.27E-69 |
| rs114036109 | 1.35E-08 | 3.10E-01 | 5.78E-02 |
| rs1260326 | 5.98E-03 | 1.54E-03 | 7.70E-92 |
| rs12898210 | 1.05E-08 | 9.63E-01 | 5.49E-02 |
| rs13107325 | 2.34E-19 | 6.30E-03 | 1.39E-03 |
| rs13306066 | 3.04E-14 | 5.89E-02 | 2.98E-01 |
| rs144503444 | 1.13E-12 | 3.53E-01 | 6.04E-10 |
| rs145947882 | 1.20E-27 | 4.84E-01 | 1.74E-11 |
| rs146035976 | 2.01E-09 | 4.08E-01 | 3.67E-01 |
| rs147233090 | 1.45E-08 | 1.79E-01 | 2.11E-11 |
| rs148933445 | 3.09E-04 | 5.50E-113 | 6.06E-04 |
| rs1561139 | 1.45E-44 | 1.13E-01 | 1.42E-02 |
| rs1671825 | 8.45E-01 | 1.83E-09 | 5.47E-03 |
| rs1716407 | 2.15E-15 | 2.64E-01 | 1.24E-06 |
| rs17248727 | 1.29E-03 | 3.98E-129 | 6.30E-01 |
| rs174418 | 6.56E-83 | 1.81E-01 | 8.07E-06 |
| rs17451107 | 7.68E-09 | 3.27E-01 | 7.84E-08 |
| rs190712692 | 3.80E-10 | 0.00E+00 | 2.07E-22 |
| rs2068888 | 5.46E-05 | 1.05E-01 | 7.13E-11 |
| rs2245365 | 3.23E-10 | 3.00E-01 | 9.43E-01 |
| rs2270925 | 1.38E-01 | 1.64E-16 | 3.00E-10 |
| rs2291956 | 1.15E-12 | 6.07E-01 | 5.79E-02 |
| rs2618568 | 9.22E-02 | 1.06E-08 | 8.01E-01 |
| rs2740488 | 1.23E-39 | 2.50E-06 | 3.95E-02 |
| rs2792751 | 1.10E-14 | 5.79E-12 | 5.55E-03 |
| rs28456 | 6.12E-25 | 3.40E-13 | 3.56E-12 |
| rs28650790 | 5.47E-07 | 1.20E-02 | 2.34E-14 |
| rs289754 | 7.24E-21 | 4.22E-02 | 4.91E-03 |
| rs2925979 | 1.79E-16 | 2.46E-01 | 3.79E-06 |
| rs2943652 | 2.15E-15 | 3.71E-01 | 1.33E-11 |
| rs3093680 | 5.64E-01 | 1.88E-01 | 5.37E-10 |
| rs34931250 | 1.64E-09 | 2.57E-02 | 1.39E-02 |
| rs34951175 | 3.42E-10 | 2.66E-02 | 5.34E-03 |
| rs35148262 | 3.37E-02 | 2.60E-09 | 7.29E-01 |
| rs35169323 | 5.04E-02 | 8.50E-03 | 1.75E-08 |
| rs35332062 | 1.66E-11 | 8.98E-01 | 5.99E-65 |
| rs3775228 | 1.13E-03 | 2.23E-02 | 3.81E-10 |
| rs3782894 | 1.81E-11 | 6.20E-02 | 4.67E-01 |
| rs3820897 | 1.02E-08 | 1.14E-01 | 2.09E-05 |
| rs3827743 | 2.95E-08 | 1.35E-02 | 1.54E-02 |
| rs41279633 | 4.69E-02 | 3.49E-15 | 2.33E-01 |
| rs4240624 | 1.15E-28 | 2.25E-12 | 5.50E-01 |
| rs4245791 | 9.68E-01 | 1.40E-42 | 6.31E-02 |
| rs4479415 | 8.87E-01 | 2.57E-08 | 1.81E-01 |
| rs4731702 | 1.73E-13 | 8.10E-01 | 3.16E-09 |
| rs4812492 | 3.20E-01 | 3.33E-12 | 4.36E-01 |
| rs4969182 | 9.08E-12 | 2.08E-06 | 3.37E-03 |
| rs56832849 | 6.59E-01 | 3.21E-14 | 1.64E-01 |
| rs5754217 | 6.32E-10 | 5.99E-02 | 1.69E-01 |
| rs59781045 | 1.46E-18 | 8.07E-02 | 6.09E-06 |
| rs59950280 | 5.87E-02 | 2.02E-05 | 1.10E-10 |
| rs6065908 | 9.30E-30 | 1.60E-02 | 7.02E-13 |
| rs6070491 | 1.36E-03 | 6.28E-01 | 4.06E-09 |
| rs61779306 | 2.14E-17 | 3.72E-01 | 6.06E-04 |
| rs61792968 | 3.53E-08 | 9.62E-01 | 3.73E-05 |
| rs62191851 | 2.73E-09 | 4.82E-01 | 6.71E-04 |
| rs629301 | 3.45E-06 | 2.10E-110 | 4.16E-01 |
| rs635279 | 2.19E-10 | 4.24E-01 | 4.98E-01 |
| rs635634 | 8.68E-04 | 3.99E-29 | 1.11E-01 |
| rs6453131 | 9.93E-01 | 1.82E-37 | 4.71E-03 |
| rs6709904 | 9.80E-01 | 3.07E-08 | 6.20E-01 |
| rs686030 | 2.71E-11 | 2.15E-01 | 6.60E-02 |
| rs693668 | 6.53E-01 | 7.70E-22 | 2.81E-01 |
| rs7134375 | 1.56E-08 | 1.38E-01 | 2.11E-04 |
| rs7140110 | 5.92E-01 | 1.48E-02 | 7.48E-10 |
| rs7219303 | 5.56E-01 | 3.37E-10 | 4.65E-01 |
| rs72786786 | 1.00E-200 | 2.71E-15 | 6.35E-05 |
| rs72926990 | 1.92E-08 | 6.16E-04 | 9.06E-01 |
| rs73002469 | 6.45E-02 | 2.03E-10 | 6.94E-01 |
| rs73013176 | 2.62E-01 | 1.95E-27 | 8.42E-01 |
| rs7395581 | 2.24E-17 | 4.69E-01 | 3.32E-10 |
| rs76259755 | 1.51E-83 | 5.52E-01 | 6.40E-124 |
| rs77303550 | 8.56E-01 | 3.25E-20 | 1.55E-03 |
| rs77619625 | 5.87E-09 | 9.59E-01 | 8.65E-02 |
| rs77960347 | 1.32E-28 | 8.77E-02 | 1.17E-01 |
| rs78357146 | 6.91E-01 | 4.98E-04 | 2.23E-08 |
| rs8060967 | 3.32E-24 | 1.04E-01 | 9.35E-04 |
| rs838878 | 3.92E-19 | 2.02E-01 | 5.34E-01 |
| rs910071 | 7.92E-02 | 4.26E-14 | 6.00E-06 |
| rs9273369 | 5.05E-03 | 6.68E-03 | 5.89E-11 |
| rs934197 | 1.20E-03 | 3.55E-73 | 5.41E-04 |
| rs9830434 | 6.14E-02 | 5.72E-09 | 6.34E-01 |
| rs987763 | 1.77E-05 | 3.64E-01 | 8.63E-11 |
| rs9980195 | 1.01E-08 | 7.00E-01 | 3.37E-01 |
