# Supplementary material for: Elevated serum LDL-C increases the risk of Lewy body dementia: a two-sample mendelian randomization study
Source: Lipids Health Dis. 2024 Feb 8;23:42. doi: 10.1186/s12944-024-02032-0 (PMC10851540; doi:10.1186/s12944-024-02032-0)
Supplement: Supplementary file 7 — Supplementary Material 7: Supplementary Table 6 Steiger direction test from LDL-C to LBD. [file 12944_2024_2032_MOESM9_ESM.docx]

**Supplementary Table 6**

Steiger direction test from LDL-C to LBD.

| R^2^.exposure | R^2^.outcome | Correct causal direction | Steiger *P* value |
| --- | --- | --- | --- |
| 0.07989177 | 0.009699774 | TRUE | 3.40E-50 |
