# Supplementary material for: Elevated serum LDL-C increases the risk of Lewy body dementia: a two-sample mendelian randomization study
Source: Lipids Health Dis. 2024 Feb 8;23:42. doi: 10.1186/s12944-024-02032-0 (PMC10851540; doi:10.1186/s12944-024-02032-0)
Supplement: Supplementary file 8 — Supplementary Material 8: Supplementary Table 7 Statistical power calculation for MR analyses. [file 12944_2024_2032_MOESM10_ESM.docx]

**Supplementary Table 7**

Statistical power calculation for MR analyses.

| Exposures | Outcome | Power |
| --- | --- | --- |
| LDL-C | LBD | 0.96 |
| HDL-C | LBD | 0.40 |
| TG | LBD | 0.22 |
