# Supplementary material for: Elevated serum LDL-C increases the risk of Lewy body dementia: a two-sample mendelian randomization study
Source: Lipids Health Dis. 2024 Feb 8;23:42. doi: 10.1186/s12944-024-02032-0 (PMC10851540; doi:10.1186/s12944-024-02032-0)
Supplement: Supplementary file 9 — Supplementary Material 9: Supplementary Table 8 IVW MR analyses after removing SNPs with secondary traits. [file 12944_2024_2032_MOESM11_ESM.docx]

**Supplementary Table 8**

IVW MR analyses after removing SNPs with secondary traits.

| Expourses | Methods | OR | 95% CI | *p* value |
| --- | --- | --- | --- | --- |
| LDL-C | UVMR | 1.323 | 1.071 - 1.635 | 0.009 |
| HDL-C |  | 0.864 | 0.718 – 1.041 | 0.124 |
| TG |  | 1.210 | 0.916 - 1.598 | 0.179 |
| LDL-C | MVMR | 1.217 | 1.002 - 1.478 | 0.048 |
| HDL-C |  | 0.843 | 0.679 - 1.046 | 0.121 |
| TG |  | 1.012 | 0.737 - 1.389 | 0.941 |

OR, odds ratio; CI, confidence interval
